# Characterization and Prevention of the Adsorption of Surfactant Protein D to Polypropylene

**DOI:** 10.1371/journal.pone.0073467

**Published:** 2013-09-11

**Authors:** Preston E. Bratcher, Amit Gaggar

**Affiliations:** 1 Department of Medicine, University of Alabama at Birmingham, Birmingham, Alabama, United States of America; 2 Gregory Fleming James Cystic Fibrosis Research Center, University of Alabama at Birmingham, Birmingham, Alabama, United States of America; 3 University of Alabama at Birmingham Lung Health Center, University of Alabama at Birmingham, Birmingham, Alabama, United States of America; 4 Medicine Service, United States Department of Veterans Affairs Medical Center, Birmingham, Alabama, United States of America; University of Tübingen, Germany

## Abstract

Surfactant Protein D (SP-D) is a multifunctional protein present in the lung and in respiratory secretions. In the process of developing new experimental approaches to examine SP-D function, we observed that SP-D adsorbs to polypropylene tubes to a great extent, thereby depleting SP-D from the solution. Although it is well known that proteins adsorb nonspecifically to plastic, this effect is usually diminished by treatments to make the plastic “low-retention” or “low-binding”. However, these treatments actually increased the binding of SP-D to the plastic. In addition, this adsorption affected the results of several assays, including proteolytic cleavage assays. In order to block SP-D from adsorbing to polypropylene and the effects caused by this adsorption, we coated the tubes with bovine serum albumin (BSA), as is commonly performed for ELISAs. This coating greatly diminished the amount of SP-D sticking to the plastic, providing an inexpensive and effective method for preventing adsorption and the artifacts resulting from this adsorption.

## Introduction

Surfactant protein D (SP-D) is a pulmonary collectin that has various roles in the lung, including surfactant homeostasis, regulation of inflammation, and innate host defense (reviewed in [1]). Its functional domains include N-terminal and collagen-like domains, which are involved in multimerization, and a carbohydrate recognition domain (CRD), which is involved in recognition of pathogens. Monomers of SP-D multimerize into trimers through the collagen-like domain, and then are able to further associate into cruciform-shaped dodecamers and even higher order multimers. Through its CRD, SP-D is able to bind to fungal, viral, and bacterial pathogens, and due to its multimeric structure, binding to these pathogens can result in the formation of aggregates, which is thought to limit pathogen invasion of host tissues as well as facilitate mucocilliary clearance and phagocytosis (reviewed in [2]).

SP-D has been found to be dysregulated in a myriad of pulmonary diseases (reviewed in [3]). While several studies have only examined levels using an ELISA format, others have shown that the structural state of SP-D is altered in many pulmonary diseases through the use of western blotting. These studies show that SP-D can be fragmented in the human lung [4], [5], and further research has described several proteases which have the ability to cleave SP-D. These proteases include host proteases such as neutrophil elastase, cathepsin G, protease 3, and, more recently, matrix metalloprotease (MMP)-9, as well as the bacterial elastase produced by *Pseudomonas aeruginosa* [6], [7], [8]. The use of SP-D as a biomarker for various diseases/severity of disease is being explored.

Over the course of our SP-D related studies, we have come across various caveats of working with SP-D. SP-D has previously been referred to as a “sticky” protein, and during our characterization of the cleavage of SP-D by MMP-9 and other experiments, we came across a confounding result that seemed to suggest that SP-D might have been strongly adsorbing to the tubes in which we were performing the cleavage reactions. The current study provides an in depth analysis of the ability of SP-D to bind to various types of tubes as well as pipet tips, and the effect this can have on experimental results.

## Results

### SP-D Adsorbs Strongly to Polypropylene

**Figure 1:**
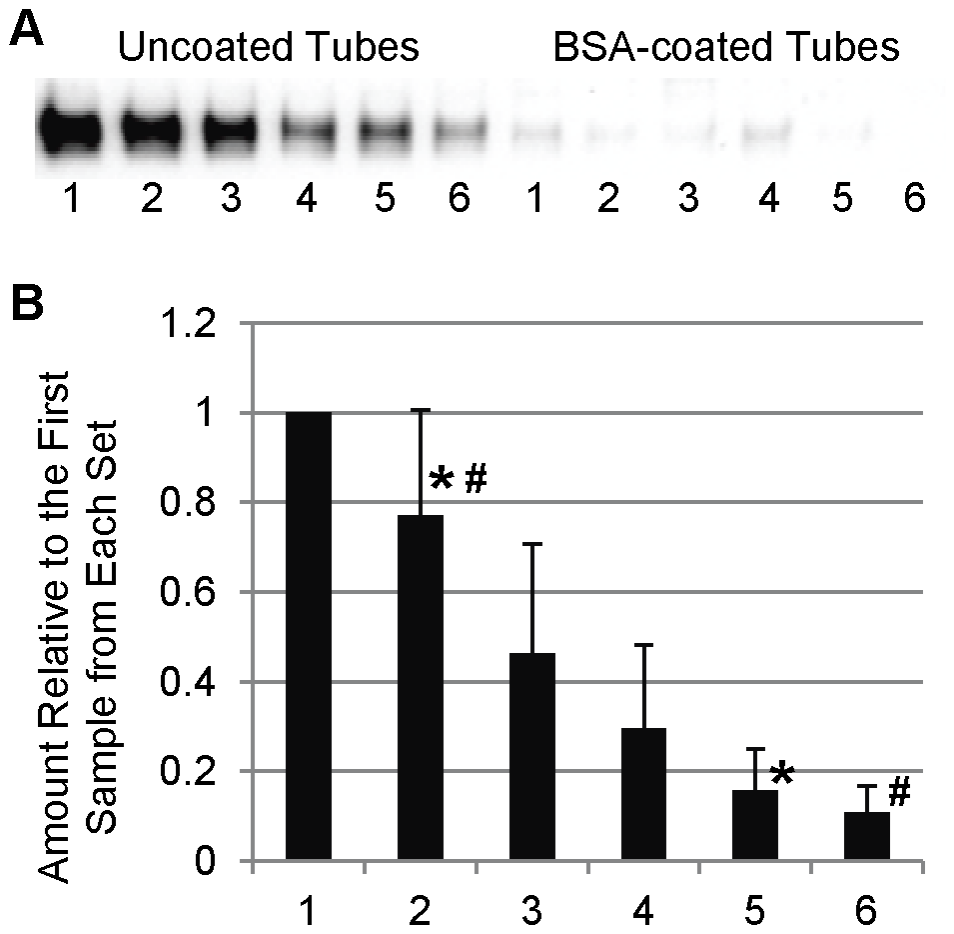
SP-D Adsorption to Polypropylene is Greatly Diminished by Coating with BSA. Western blotting detects the 43-D stripped from polypropylene tubes using Laemmli Buffer (A). 1 through 6 denotes the tube order during serial transfer. The amount of SP-D detected on uncoated tubes was quantified using ImageJ software (B). Columns with an asterisk (*) are significantly different (p≤0.05), as are columns labeled with the number sign (#, p≤0.01).

**Figure 2:**
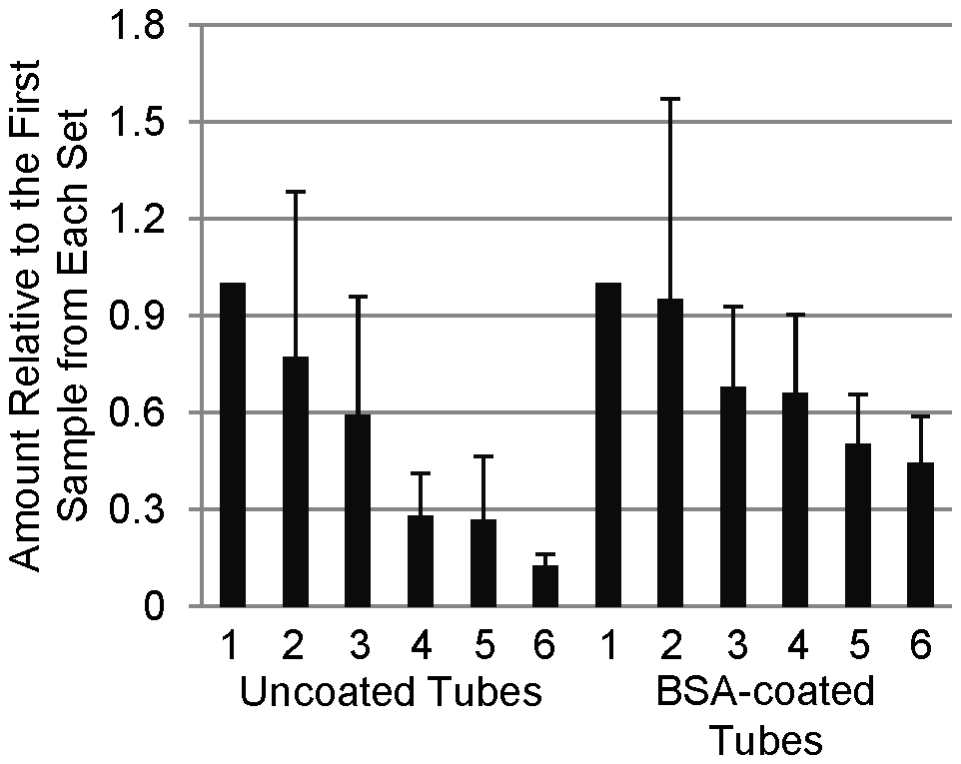
Depletion of SP-D in Solution During Serial Transfers. 5 µl aliquots were taken from a serially transferred solution of SP-D after each incubation, analyzed by Western blot, and quantified using ImageJ software.

**Figure 3:**
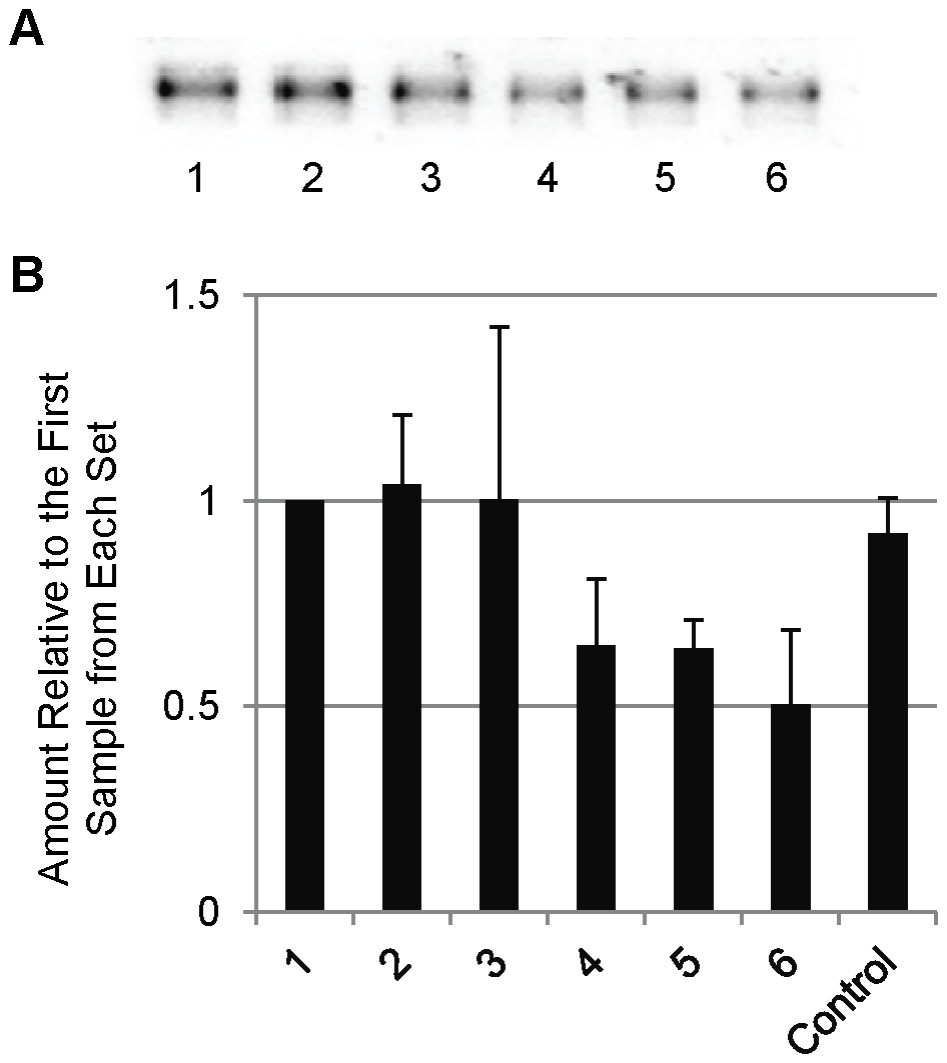
Adsorption of SP-D to Polypropylene Pipet Tips. 5 µl aliquots were taken from a serially aspirated/dispensed solution of SP-D after each round of pipetting, analyzed by Western blot (representative blot shown in A), and quantified using ImageJ software (B). The control consisted of 6 aliquots taken without pipetting. The means were found to be significantly different by One-way ANOVA (p = 0.044).

**Figure 4:**
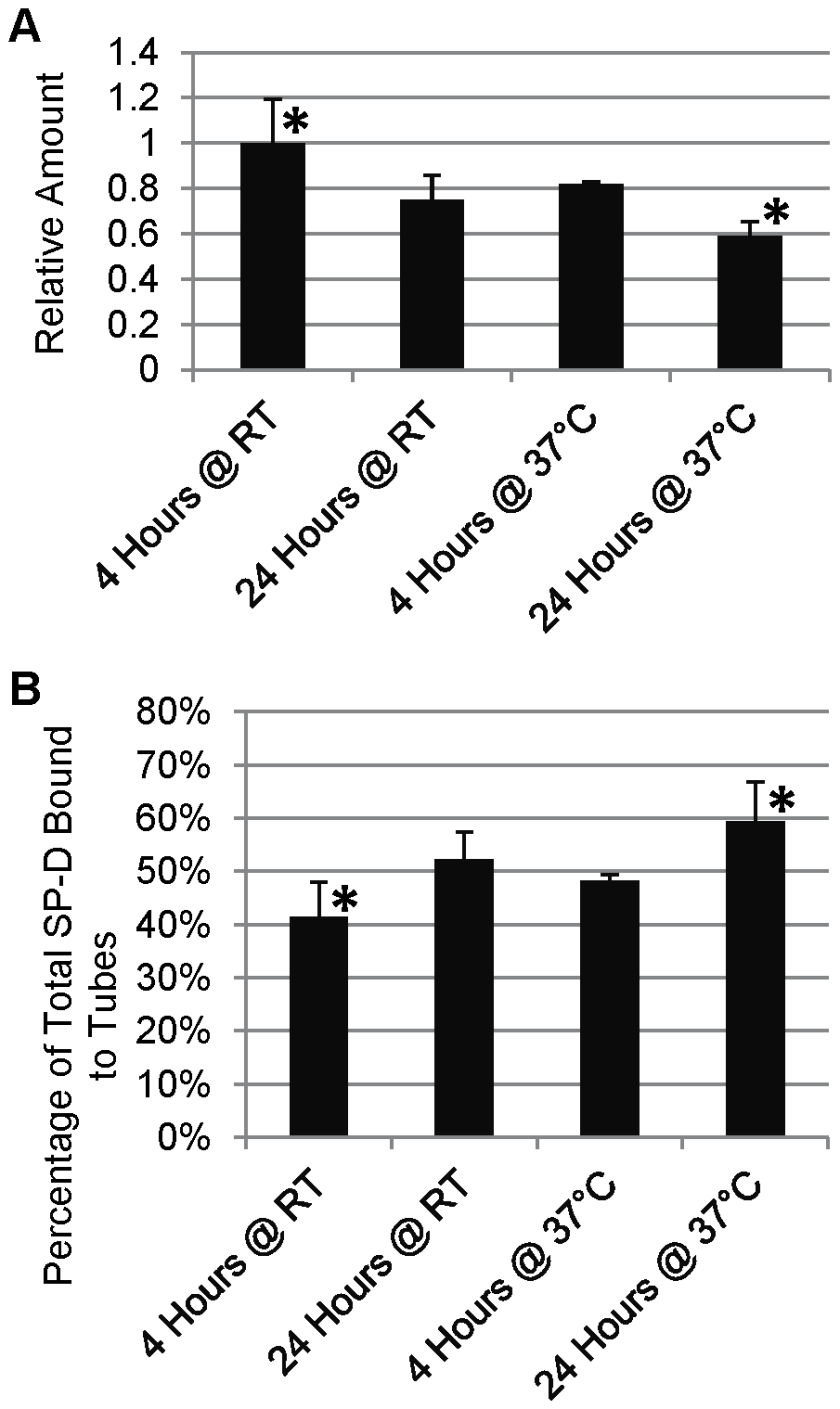
Depletion of SP-D during Extended Incubations. 5 µl aliquots were taken from SP-D solutions after incubating at the indicated time/temperature, analyzed by Western blot, and quantified using ImageJ software (A). Values were normalized to the average of the “4 hours @ RT” group. The SP-D content of tubes was analyzed and compared to the total amount of SP-D in the supernatant and tube (B). An asterisk (*) denotes p≤0.05 when comparing columns.

We examined the adsorption of SP-D to polypropylene tubes by incubating tubes for 10 minutes with a 1 µg/mL SP-D solution. After transfer of the solution to a clean tube, the tube was washed and SP-D was stripped from the tube by boiling the tube with reducing Laemmli buffer. This process was repeated 5 times, for a total of 6 tubes for each set of transfers (Figure 1). A noticeable decrease in the amount of SP-D detected was seen with each subsequent transfer. In order to determine whether this was due to a decrease in the SP-D content of the solution, we examined the levels of SP-D in the supernatant of the solutions after incubations in the tubes (Figure 2). Although there was extensive variation amongst the sample sets, each set revealed decreases in detectable SP-D levels in solution after incubation in each subsequent tube, resulting in a very considerable decrease between the first and last samples of each set. As the pipet tips we used are also made of polypropylene, we examined the extent of SP-D binding to the tips during aspiration and transfer of the samples (Figure 3). In order to do this, we repeated the transfer experiment as described above, except instead of transferring to a clean tube, the solution was reapplied to the same tube, with an aliquot removed after each incubation/aspiration/dispensing. As a control, aliquots were removed without any pipetting. A measureable decrease in SP-D levels was observed as a result of pipetting the solution. To examine the extent that SP-D could be depleted from the samples by adsorption, we examined the effect of longer incubations times along with incubation at 37°C. As shown in Figure 4A, the amount of SP-D in solution was decreased in a time and temperature-dependent manner. After measuring the amount bound to the tubes in the same experiment, we were able to calculate the percentage of total SP-D that had adsorbed to the tubes (Figure 4B). This result demonstrates that during the conditions in which we have previously performed some experiments (1 µg/mL SP-D incubated for 24 hours at 37°C), around 60% of the SP-D would have been adsorbed to the polypropylene tube.

### Commercially Available Tubes to Reduce Adsorption Increase the Adsorption of SP-D

**Figure 5:**
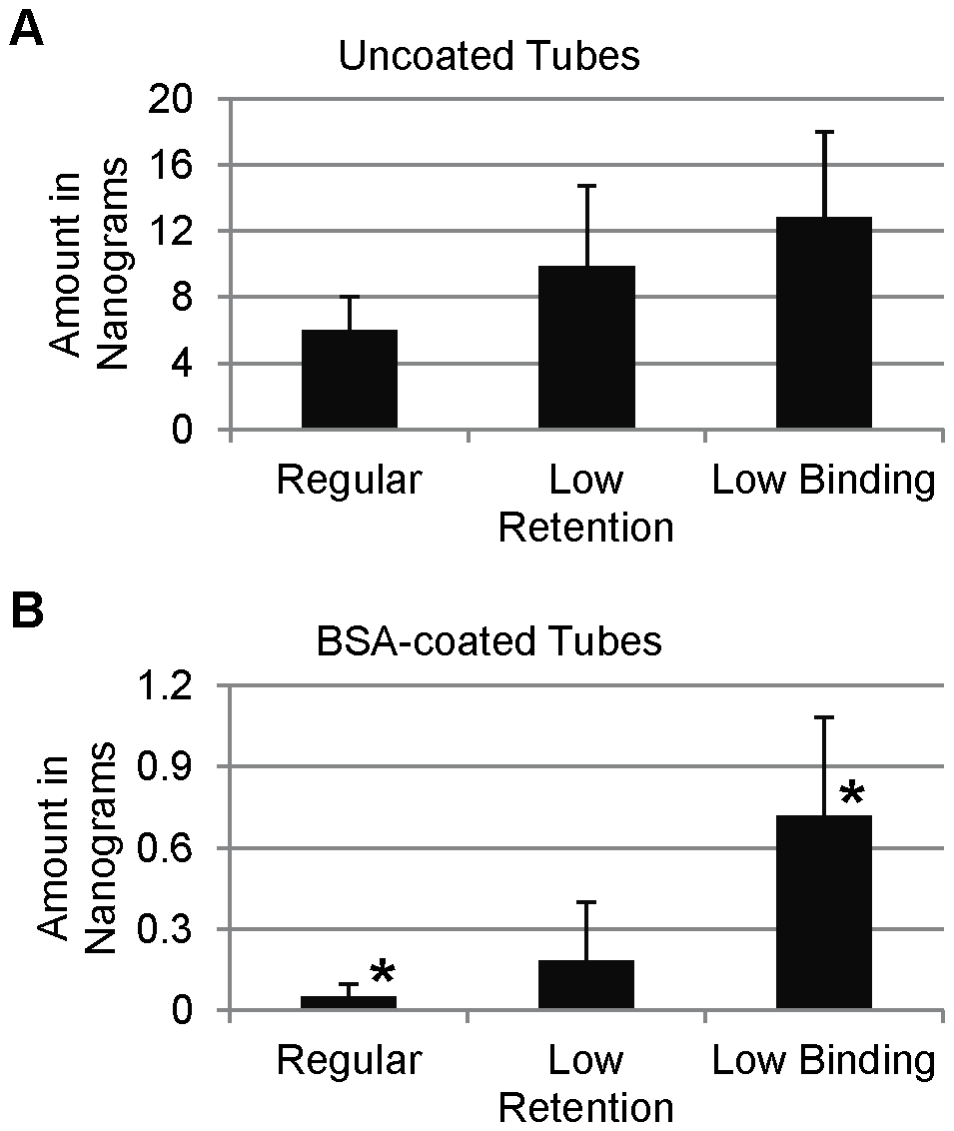
Adsorption of SP-D to Variously Treated Polypropylene Tubes. The amount of adsorbed SP-D was measured using ImageJ software to analyze Western blots of samples taken from uncoated (A) or BSA-coated (B) regular, low retention, and low binding tubes. The amount was calculated using untreated SP-D as a standard. An asterisk (*) denotes p≤0.05 when comparing columns.

In order to see if adsorption to this extent was unique to SP-D, we tested various other purified proteins using these same techniques. To our surprise, these proteins did adsorb similarly to SP-D in polypropylene tubes (data not shown). It is well established that proteins adsorb to polypropylene (just as in the coating of ELISA plates), and as a result, manufacturers have made polypropylene tubes [termed “low retention” or “low binding” (see Methods)] available that have been treated in order to decrease this adsorption. When we tested these tubes in the conditions used to generate the data for Figures 1 and 2, we found that these tubes adsorbed more SP-D compared to the untreated polypropylene tubes (Figure 5A). As a control, we tested another protein (mouse IgG1-HRP conjugate) in these same conditions, and observed a significant decrease in adsorption [∼50% reduction of adsorption in low retention tubes (compared to regular tubes) and a ∼85% reduction of adsorption in low binding tubes (data not shown)]. Although it was not tested, we imagine the use of these treated tubes in the conditions used for Figure 4 would result in an even greater depletion of SP-D from the solution. This demonstrates a unique property of SP-D, and we therefore sought other methods to reduce the adsorption of SP-D to polypropylene.

### Coating Tubes with BSA Greatly Diminishes SP-D Adsorption

Due to its use as a blocking agent in ELISAs, we first decided to test the ability to block adsorption of SP-D by coating the polypropylene tubes with BSA. Various conditions were explored to optimize the coating of tubes with BSA to block this adsorption, and we determined the optimal coating conditions to be treatment with 100 mg/mL BSA in dH_2_O for 24 hours (Figure S1). Using these conditions, we compared the levels of adsorption by untreated polypropylene tubes to those coated with BSA (Figure 1A). BSA-coating resulting in a very substantial decrease in the amount of SP-D detected on the tubes. We next examined the amount of SP-D remaining in solution after the serial transfer experiment, and we found that we were able to detect higher levels of SP-D in the BSA-coated tubes (Figure 2). We attribute the loss of detectable SP-D in the BSA-coated tubes to adsorption to the pipet tips used for transfer of the solution, as shown in Figure 3. Interestingly, when we applied this BSA-coating technique to the low retention and low binding tubes, both tubes adsorbed considerably less SP-D than without BSA coating, but still had higher detectable levels than the BSA-coated untreated polypropylene tubes (Figure 5). We therefore conclude that, of the methods we tried, the most successful for decreasing SP-D adsorption to polypropylene is to coat untreated polypropylene with BSA.

### Adsorption of SP-D to Polypropylene Changes the Kinetics of Cleavage by MMP-9

**Figure 6:**
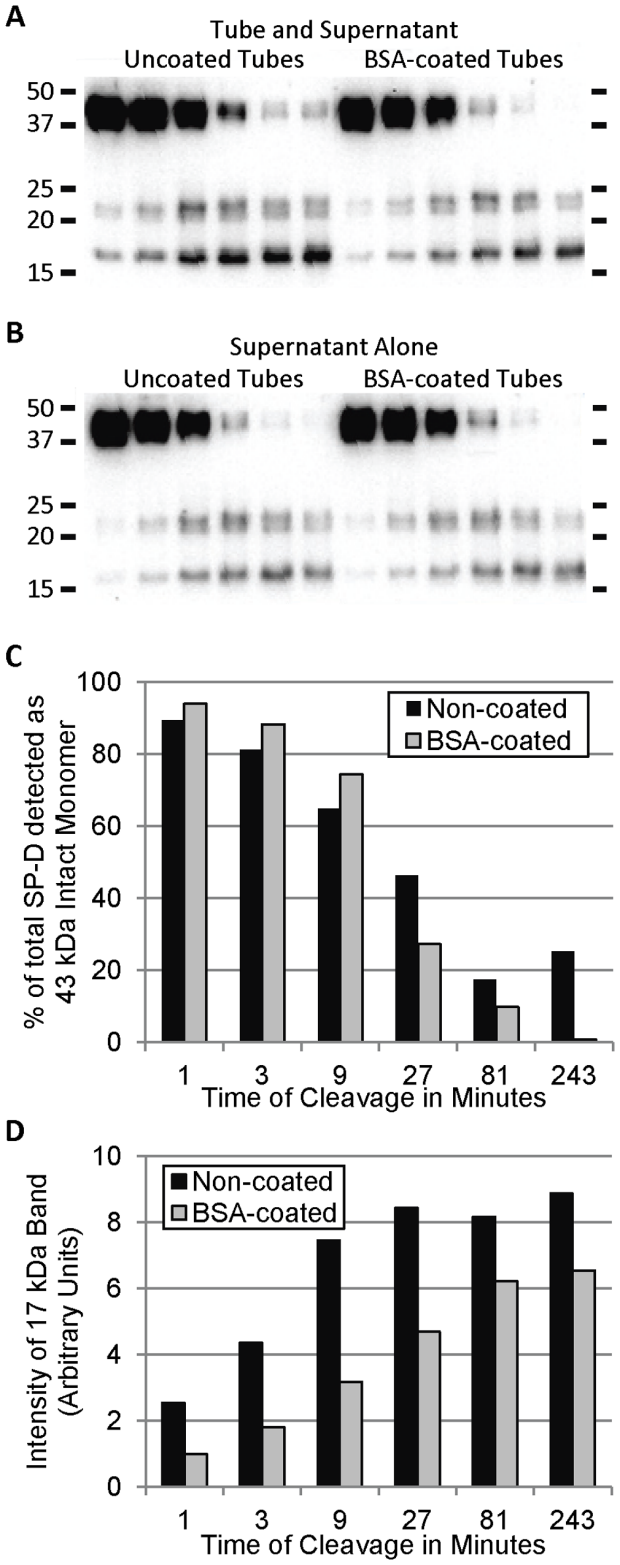
MMP-9-mediated Cleavage of SP-D is Affected by Adsorption to Polypropylene Tubes. Western blots of cleaved SP-D from either tubes and supernatants combined (A) or from supernatants alone (B). Cleavage was performed in both uncoated (first 6 lanes) and BSA-coated (last 6 lanes) tubes, and samples were analyzed after incubations for 1, 3, 9, 27, 81, and 243 minutes at 37°C. For (C) and (D), bands from the Western blot shown in (A) were quantified using ImageJ software. Western blots are representative of three independent experiments.

One of the observations that clued us into the fact that SP-D was being adsorbed to polypropylene occurred during our MMP-9-mediated cleavage experiments. In these experiments, we saw different rates of fragment generation dependant on whether we took aliquots of the reaction mixture or if we examined the entire tube contents (uncoated tubes in Figure 6A vs. 6B). While SP-D examined in the supernatants from either uncoated or coated tubes was able to be completely cleaved under the conditions used, around 20% of the SP-D in the uncoated combined tube and supernatant portion remained intact (Figure 6C). In addition, the rate of generation of the 17 kDa fragment was faster in the combined tube and supernatant portion (Figure 6D). Additionally, although the kinetics of generation of a specific fragment did not change, the kinetics of loss of the intact band during cleavage with NE was also changed due to adsorption of SP-D to the tubes (Figure S2). With both the MMP-9 and NE-cleavage reactions, the differences seen between the supernatant and the tube/supernatant could be eliminated using BSA-coated polypropylene tubes.

## Discussion

Much work has been done to elucidate the functions and properties of SP-D, and this continues to be an active arena of research. The ability of SP-D to bind to treated polypropylene tubes as described in this study has the potential to cause experimental artifacts. Fortunately, this adsorption is largely attenuated by using the ELISA-like technique of blocking with BSA. It is important to note that adsorption at the levels/rates described in this study is only applicable to situations in which purified SP-D is used in a solution with little or no other protein. Although precoating polypropylene with BSA allows one to examine purified SP-D in this context, the presence of BSA in solution also greatly reduces the adsorption of SP-D (Figure S1C). We suggest that the presence of other proteins in complex samples, such as lavage fluid and sputum, may prevent the majority of SP-D adsorption to the polypropylene surface of tubes.

Our lab has already used this technique of coating polypropylene with BSA to eliminate experimental artifacts that we had previously witnessed in our studies. For example, we were interested in examining if ligand binding by the CRD could alter the multimeric structure of SP-D, as differences in order of multimerization has previously been reported [9], [10], [11]. To study this, we performed Western blots using native PAGE of SP-D incubated with or without *E. coli* LPS for 24 hours at 37°C in low-binding tubes. Initial blots showed a LPS dose-dependent increase in the amount of SP-D visualized, with almost no SP-D seen without LPS (data not shown). As the different multimeric structures of SP-D are present in different amounts, increasing the LPS (which increased the amount of SP-D recovered), resulted in the appearance of unique bands. With additional experiments (including repeating this method in BSA-coated tubes), however, we were able to determine the source (artifact) causing these results, leading to the present study. We have also provided evidence that the adsorption of SP-D can cause other unexpected variations in experimental results, as shown in our MMP-9 cleavage assay, and using BSA-coated tubes abrogated this effect (Figure 6).

These findings suggest that there may be a large variation in the precise concentrations of SP-D used in experimental systems. During the process of preparing and transferring SP-D-containing solutions for an experiment, it is highly likely that the actual concentration is being altered due to adsorption to the materials used. Therefore, working concentrations reported in the literature may not be accurate, although the biological effects of SP-D should be relevant. Although it may be impractical to monitor concentrations of exogenously added SP-D at various times throughout an experiment, it seems prudent to prepare reagents in a consistent manner.

This study highlights the possibility of experimental artifacts due to the adsorption of SP-D to polypropylene. Adsorption had a dramatic effect on measureable SP-D levels, and even the number of times a solution is pipetted significantly affected the SP-D levels. Importantly, the polypropylene tubes that were treated specifically to lower protein adsorption actually bound more SP-D than untreated tubes. In this study, we demonstrate that coating the tubes with BSA greatly diminishes the adsorption of SP-D, thereby preventing the artifact-causing effects we had previously detected in our experiments.

## Materials and Methods

### Western Blots

SP-D was visualized by reducing SDS-PAGE using 15% acrylamide gels. Proteins were transferred to a nitrocellulose membrane and blocked with 5% milk at 4°C overnight. Membranes were incubated with primary (AF1920 diluted 1∶2,000, R&D Systems, Minneapolis, MN, USA) and then secondary (A9452 diluted 1∶40,000, Sigma, St. Louis, MO, USA) antibodies for one hour each at room temperature (RT), with washing after each incubation. Blots were imaged using Supersignal West Femto (Thermo, Waltham, MA, USA) and a ChemiDoc XRS (BioRad, Hercules, CA, USA). Intact monomers of SP-D are visualized at ∼43 kDa.

### SP-D Depletion by Pipetting

Purified recombinant human SP-D (R&D Systems, Minneapolis, MN, USA) was diluted to a concentration of 1 µg/mL in DPBS containing calcium and magnesium (Thermo Scientific, Waltham, MA, USA). 100 µl of this solution was added to each tube or to the initial tube in the serial transfer experiments. Either normal polypropylene (Fisher, Hampton, NH, USA), siliconized “low retention” polypropylene (Fisher, Hampton, NH, USA), or prelubricated “low binding” polypropylene (Corning, Tewksbury, MA, USA) 1.5 mL tubes were used. For all experiments, polypropylene pipet tips (Fisher, Hampton, NH, USA) were used. To analyze SP-D content in the supernants, 5 µl samples were removed, mixed with an equal volume of Laemmli buffer (BioRad, Hercules, CA, USA) containing β-mercaptoethanol (Sigma, St. Louis, MO, USA), boiled, and visualized by Western blot. To analyze SP-D adsorbed to the tubes, all remaining solution was aspirated and the tube was washed twice with 1 mL of DPBS with calcium and magnesium. The tubes were then flash spun and the remaining solution was again aspirated. 30 µl of Laemmli buffer containing β-mercaptoethanol was added, each tube was thoroughly vortexed before and after boiling, and SP-D was then visualized by Western blot. In the serial transfer experiments, the SP-D solution was incubated for 10 minutes at room temperature in the tube, a 5 µl sample was removed, and then the remaining solution was transferred to a new tube. This process was then repeated.

### BSA coating of polypropylene tubes

BSA (Sigma, St. Louis, MO, USA) was dissolved in deionized water at a concentration of 100 mg/mL, and then sterile filtered. 100 µl of the BSA solution (or water in the uncoated samples) was incubated in the 1.5 mL tubes for 24 hours at room temperature. The solution was then aspirated, and the tubes were washed twice with 1 mL of DPBS containing calcium and magnesium, flash spun, and all remaining solution was aspirated.

### SP-D Cleavage by MMP-9 and NE

SP-D cleavage assay were performed as previously reported [7]. Briefly, 5 µl reactions containing 20 µg/mL of recombinant SP-D were incubated at 37°C in polypropylene tubes. At the indicated time, 2.5 µl was removed and added to a tube containing 2.5 µl Laemmli buffer (supernatant sample) and then 2.5 µl of Laemmli buffer was added to the remaining 2.5 µl of the reaction volume in the reaction tube (tube sample). Samples were then boiled and visualized by Western blot. For the MMP-9 cleavage assays, active recombinant human MMP-9 (Millipore, Billerica, MA, USA) at 60 nM was used with DPBS with calcium and magnesium as the assay buffer. For the NE cleavage assays, purified NE (Millipore, Billerica, MA, USA) at 15 nM was used with 100 mM Tris-HCl (pH 7.5), 500 mM NaCl as the assay buffer.

### Analysis of Western Blots

In order to quantitate the relative intensity of bands on Western Blots, the captured images of ECL-developed blots were subject to analysis using ImageJ (Rasband, W.S., ImageJ, U. S. National Institutes of Health, Bethesda, Maryland, USA, http://imagej.nih.gov/ij/, 1997–2012). After relative intensities were established, these values were normalized to the intensity of the band from the first tube (for Figures 1, 3, and 4) or to a 5 ng standard that was loaded in duplicate (Figure 2). In order to calculate the percentage of total SP-D that was stuck to a given tube, the relative intensity between the supernatant and the corresponding tube was established using ImageJ, the value assigned to the supernatant was multiplied by 20 (since 5 µl of 100 µl total was subjected to Western blotting), and the tube value was divided by the tube+corrected supernatant value (Figure 5B). For all other values, relative intensities calculated by ImageJ were normalized according to the corresponding figure legend.

### Statistics

In the figures for which statistical analysis was performed, all samples were run in triplicate, and results shown are representative of at least 3 independent experiments. One-way ANOVA followed by Tukey’s multiple comparisons test was performed using GraphPad Prism version 5.00 for Windows (GraphPad Software, La Jolla California USA). In all graphs, the error bars denote the standard deviation of the data.

## Supporting Information

Figure S1 **Optimization of Conditions for Coating with BSA.** Silver staining was used to detect BSA adsorbed to tubes incubated for various times with various concentrations of BSA (A). For (B), tubes were incubated with a solution of BSA at the indicated concentration for 24 hours, washed, incubated with 5 µg/mL SP-D for 10 minutes, washed again, and SP-D was removed for analysis by Western blot. For (C), uncoated tubes were incubated with a solution containing 5 µg/mL SP-D and varying concentrations of BSA (four fold serially diluted from the highest concentration) for 10 minutes, washed, and SP-D content of the tubes was analyzed by Western blot as previously described. For all panels, the concentrations listed is for the BSA in the solution used.
(TIF)

Figure S2 **NE-mediated Cleavage of SP-D is Affected by Adsorption to Polypropylene Tubes.** Western blots of cleaved SP-D from either tubes and supernatants combined or from supernatants alone. Cleavage was performed in both uncoated and BSA-coated tubes, and samples were analyzed after incubations for 27, 81, and 243 minutes at 37°C. Western blots are representative of three independent experiments.
(TIF)
