# Supplementary figures and images for: Characterization and Prevention of the Adsorption of Surfactant Protein D to Polypropylene
Source: PLoS One. 2013 Sep 11;8(9):e73467. doi: 10.1371/journal.pone.0073467 (PMC3770593; doi:10.1371/journal.pone.0073467)

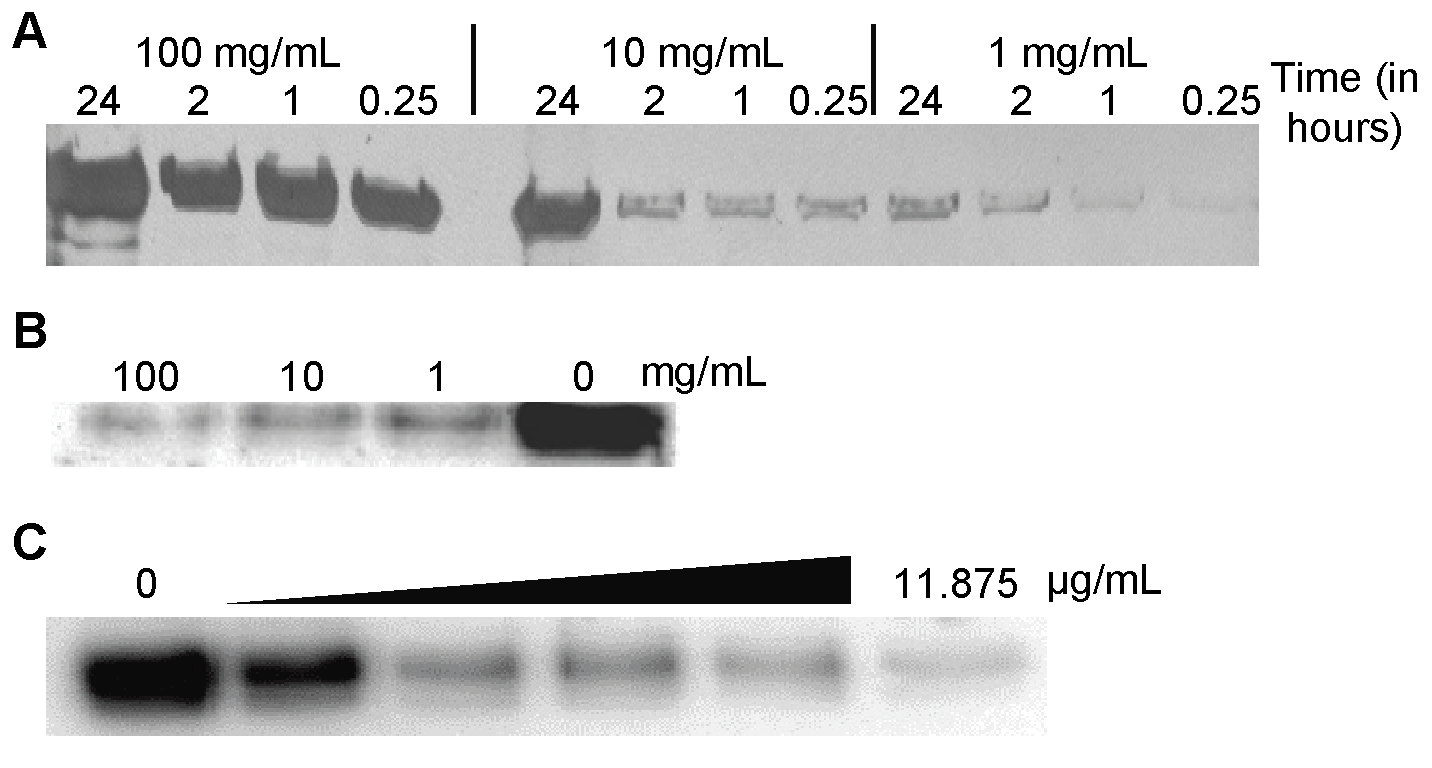

Supplement: Figure S1 — Optimization of Conditions for Coating with BSA. Silver staining was used to detect BSA adsorbed to tubes incubated for various times with various concentrations of BSA (A). For (B), tubes were incubated with a solution of BSA at the indicated concentration for 24 hours, washed, incubated with 5 µg/mL SP-D for 10 minutes, washed again, and SP-D was removed for analysis by Western blot. For (C), uncoated tubes were incubated with a solution containing 5 µg/mL SP-D and varying concentrations of BSA (four fold serially diluted from the highest concentration) for 10 minutes, washed, and SP-D content of the tubes was analyzed by Western blot as previously described. For all panels, the concentrations listed is for the BSA in the solution used. (TIF) [file pone.0073467.s001.tif]

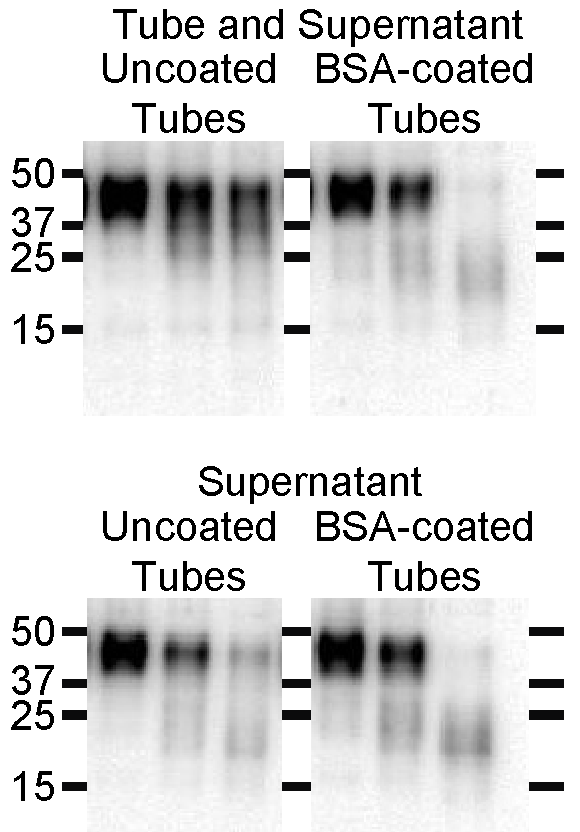

Supplement: Figure S2 — NE-mediated Cleavage of SP-D is Affected by Adsorption to Polypropylene Tubes. Western blots of cleaved SP-D from either tubes and supernatants combined or from supernatants alone. Cleavage was performed in both uncoated and BSA-coated tubes, and samples were analyzed after incubations for 27, 81, and 243 minutes at 37°C. Western blots are representative of three independent experiments. (TIF) [file pone.0073467.s002.tif]
